# Optimization of Semen Extender for Mongolian Sheep by Integrating Metabolomics and Sperm Quality Analysis at 17 °C with Antioxidant Supplementation

**DOI:** 10.3390/ani16162508

**Published:** 2026-08-12

**Authors:** Zhilei Li, Fan Zhu, Yanyun Zi, Zhenyu Gao, Min Zhao, Qinglin Yang, Hao Wu, Haipeng Zhang, Qi Jiao, Chunying Zhang, Rongtao Wang, Xiyan Li, Fangxin Zhao, Yanhui Shi, Tao Li, Shenyuan Wang, Yiyi Liu, Lu Li, Fanhua Meng, Junwei Cao, Wenguang Zhang, Xiaolong He, Shaoyin Fu, Dayong Chen, Chunxia Liu, Yongbin Liu

**Affiliations:** 1 Inner Mongolia Autonomous Region Key Laboratory of Bio-Manufacturing Technology, Inner Mongolia Industrial Technology Engineering Center of Livestock Novel Germplasm Material Creation, Inner Mongolia Industrial Technology Engineering Center of Agricultural Genomic Big Data, Inner Mongolia Regional Speciality Livestock Biotechnology Innovation Team, College of Life Sciences, Inner Mongolia Agricultural University, Hohhot 010018, China; lizhilei2002@163.com (Z.L.); 18304905355@163.com (F.Z.); ziyanyun173153@163.com (Y.Z.); 13664776097@163.com (Z.G.); zhaomin19972024@163.com (M.Z.); yangql0305@163.com (Q.Y.); 15249464965@163.com (H.W.); zhpzhzhs@gmail.com (H.Z.); 15148293759@163.com (Q.J.); 15149130480@163.com (C.Z.); 13190602002@163.com (R.W.); 15647188082@163.com (X.L.); 15847860670@163.com (F.Z.); shiyanhui2020222@163.com (Y.S.); 15904810962@163.com (T.L.); wsy1688521@126.com (S.W.); 15104718293@139.com (Y.L.); yunsong-5410@163.com (L.L.); fhmeng2013@126.com (F.M.); caojunwei1970@163.com (J.C.); wenguangzhang@imau.edu.cn (W.Z.); 2 Inner Mongolia Academy of Agricultural & Animal Husbandry Sciences, Hohhot 010031, China; hexiaolong1983@163.com (X.H.); fushao1234@126.com (S.F.); 3 Inner Mongolia Sino Breeding Sheep Technology Co., Ltd., Ulanqab 011800, China; chendayong81@126.com; 4 China-Mongolia Biomacromolecule Application “Belt and Road” Joint Laboratory, Hohhot 010018, China; 5 Key Laboratory of Mutton Sheep Genetics and Breeding, Ministry of Agriculture and Rural Affairs, Hohhot 010018, China; 6 College of Animal Science, Inner Mongolia Agricultural University, Hohhot 010018, China

**Keywords:** Mongolian sheep, sperm, diluent, 17 °C, sperm quality, metabolomics

## Abstract

This study focuses on optimizing the liquid preservation protocol for Mongolian sheep semen at 17 °C. We systematically compared the protective effects of eight extender formulations on sperm quality and analyzed dynamic metabolic changes under optimal storage conditions via LC-MS/MS metabolomics, aiming to provide experimental evidence for improving semen preservation techniques and to establish a foundation for the selection of preservation media in future germplasm conservation efforts. By comparing eight diluents, a semen preservation formulation for Mongolian sheep was optimized. The eighth diluent, including fructose, lactose, and Tris, effectively sustained sperm motility during liquid storage at 17 °C. To clarify the underlying metabolic mechanism, LC-MS/MS metabolomics identified 2669 metabolites in spermatozoa at storage times of 0, 48, 96, and 144 h. Based on the metabolomics analysis of differential metabolites, three antioxidants including vitamin E (acting as the basic antioxidant protection), lycopene (for targeted inhibition of lipid peroxidation) and resveratrol (suggested to have endogenous protective effects) were used for exogenous addition experiments. Subsequently, the optimal concentrations for addition were screened: resveratrol (25 μM), lycopene (5 μM), and vitamin E (1.5 μM). Verification experiments revealed that each of the three antioxidants could independently sustain sperm viability above 50% for up to 7 days. Moreover, the combined supplementation exhibited a synergistic effect, increasing sperm viability to 55.05%. This study not only elucidates the underlying mechanism of sperm preservation at the metabolic level, but also provides a validated formulation basis and theoretical support for the development of efficient sheep semen preservatives.

## Simple Summary

This study aimed to optimize liquid storage of Mongolian sheep semen at 17 °C, supporting artificial insemination and genetic conservation. Eight diluents were tested to identify an optimal formula that maintained sperm motility for extended periods. To explore the underlying changes, metabolomic analysis of spermatozoa was performed at 0, 48, 96, and 144 h. Among the differential metabolites, resveratrol was predicted to exert potential endogenous protective effects. Based on this metabolomic clue and known antioxidant mechanisms, the diluent was supplemented with vitamin E (as a general antioxidant) and lycopene (to specifically counter lipid peroxidation) at screened concentrations. Individually, all three antioxidants sustained sperm viability above 50% for up to 7 days. When combined, they showed synergistic action, raising viability to 55.05%. These findings offer mechanistic insights into sperm preservation and provide a practical, evidence-based formulation for the optimization of sheep semen extenders.

## 1. Introduction

The Mongolian sheep (*Ovis aries*), one of the most widely distributed sheep breeds in China, is a vital livestock resource on the Mongolian Plateau. Ongoing modernization of animal husbandry renders improving reproductive efficiency and conserving genetic resources of Mongolian sheep critical industry objectives. Artificial insemination, a key technology in modern animal husbandry, relies heavily on efficient sperm preservation methods. The efficiency of semen preservation directly determines the retention of genetic traits, the optimization of population structure, and the overall enhancement in reproductive performance of Mongolian sheep [1]. Currently, semen preservation is achieved through three principal methods: ambient temperature (15–25 °C), refrigeration (∼4 °C), and cryopreservation (−196 °C) [2,3,4]. The cryopreservation of sperm in liquid nitrogen is widely adopted in practice. Nevertheless, sheep sperm is temperature-sensitive and susceptible to injuries induced by ice crystal formation, oxidative stress, and osmotic pressure alterations during freezing, resulting in significant declines in sperm motility, membrane integrity, and DNA quality. Improper cooling or thawing procedures may further exacerbate such damage, leading to the irreversible impairment of sperm function and fertilization capacity [5,6]. Therefore, liquid state storage serves as a viable alternative and has emerged as the predominant option in both ovine production research and field applications.

Research demonstrates that ram semen can be stored in liquid form at 4–25 °C, and sperm motility and fertility decrease markedly with rising storage temperature [7]. Elevated storage temperatures accelerate sperm metabolism, leading to rapid functional deterioration. While traditional low-temperature storage (e.g., 4–5 °C) extends sperm viability, it carries risks of cold shock and imposes stringent requirements on diluent composition and handling protocols [8,9]. Under such circumstances, storage at approximately 17 °C has emerged as a promising compromise: it slows sperm metabolism and alleviates cold stress injuries incurred during refrigeration storage. Its alignment with the ambient temperature during the natural breeding seasons of Mongolian sheep (spring and autumn) makes it a practical reference temperature for semen transport and short-term storage. However, systematic research on the liquid preservation systems of Mongolian ram semen at 17 °C remains limited, particularly regarding the optimization of diluents tailored to this specific temperature. Even under optimal low-temperature conditions, sperm quality declines over time, mainly attributable to oxidative damage, dysregulated energy metabolism, microbial contamination, and the accumulation of metabolic byproducts [10,11]. Accordingly, optimizing semen extender formulations to construct a stable in vitro micro-environment for sperm is crucial for enhancing preservation efficacy. Therefore, day 7 was selected as a field-relevant endpoint, as the interval from semen collection to insemination in routine production typically spans several days, reflecting the typical turnaround window for semen distribution and use.

Achieving efficient preservation at 17 °C requires rational design of extender compositions. Conventional semen extenders generally encompass the following functional roles. For instance, energy sources (e.g., fructose, glucose) supply substrates for sperm metabolism [12]. Additionally, buffering systems (e.g., Tris, sodium citrate) are employed to sustain an optimal pH environment (approximately 6.5–7.2), thereby decelerating metabolic rates and extending survival time [13,14]. Furthermore, antibacterial agents (e.g., penicillin, streptomycin) inhibit bacterial proliferation; however, concerns over cytotoxicity and antibiotic resistance have stimulated research into alternatives such as antimicrobial peptides [15]; Antioxidants (e.g., superoxide dismutase, glutathione, vitamins E/C) counteract oxidative stress to protect sperm membrane and DNA integrity [16]; Furthermore, protective agents (e.g., egg yolk, bovine serum albumin) stabilize sperm membrane structure under hypothermic conditions and provide supplementary nutrition [17,18].

Although the roles of extender components (e.g., energy substrates, antioxidants, ions) have been well documented in practical applications, their dynamic and molecular regulatory effects on sperm metabolism during storage remain poorly understood, with most formulations optimized empirically [19]. In recent years, the integration of metabolomics technology has offered a novel perspective for addressing this bottleneck. As a pivotal element of the omics technology, metabolomics utilizes spectroscopic and analytical techniques to comprehensively profile small-molecule metabolites in biological samples. These metabolites, being the end-products of downstream gene expression, provide a more direct and genuine reflection of cellular physiological states [20]. In reproductive research, metabolomics facilitates improvements in semen preservation strategies by identifying biomarkers, elucidating injury mechanisms, and guiding the optimization of extenders and additives [21]. In human studies, specific seminal plasma metabolites have been identified as closely linked to sperm physiological function and fertility disorders [22]. Similarly, studies on livestock such as cattle indicate that certain seminal plasma metabolites can act as biomarkers for in vivo fertility [23]. Metabolomic information enables the shift in extender formulation from empirical addition to mechanism-based design

Based on this context, the present study is designed to systematically compare the effects of various extender formulations on sperm quality in Mongolian sheep at 17 °C. Through the integration of LC-MS/MS metabolomics technology, an in-depth analysis will be performed on the dynamic changes in the sperm metabolome under optimal storage conditions. The role of key differential metabolites in semen preservation will be validated functionally. The work seeks to establish a foundation for developing highly efficient and stable liquid preservation media for Mongolian ram semen. Furthermore, it will provide theoretical insights for sperm preservation research in related livestock species.

## 2. Materials and Methods

### 2.1. Preparation of Semen Basal Extenders

According to the formulation listed in Table 1, each constituent was accurately weighed and transferred into a beaker. Distilled water was added, and the mixture was stirred until all solids were fully dissolved. The solution was transferred into a volumetric flask and diluted to a final volume of 100 mL. The solution was mixed thoroughly by repeated inversion to obtain a homogeneous mixture.

### 2.2. Semen Collection

The estrous ewes were transported to the semen collection room and fixed on dedicated racks to entice the breeding rams. Following this, the breeding rams scheduled for semen collection were moved to the waiting area adjacent to the collection room and processed individually in sequence. The operator, holding the artificial vagina and semen collection cup, swiftly led each ram out of the collection area upon completion of ejaculation. The remaining rams were subjected to identical collection procedures. (Collections were performed three times weekly, from 8:00 to 8:30 a.m.)

Semen samples were collected from six healthy Mongolian rams (aged 36 months, with a body weight of 65–85 kg) maintained at Inner Mongolia Jinlai Animal Husbandry Technology Co., Ltd. (Ulanqab, China). All rams underwent clinical examination and were confirmed to be free of reproductive diseases and other infectious disorders, with normal libido and satisfactory semen quality according to official breeding records. During the experiment, the rams were fed a concentrate-based diet supplemented with corn kernels, carrots, and eggs (1–2 per ram), with water ad libitum. Semen collections were performed three times per week (Monday, Wednesday, and Friday) from 8:00 to 8:30 a.m. All animal procedures were approved by the Animal Ethics Committee of Inner Mongolia Agricultural University (Approval No. NND2024127) and conducted in accordance with institutional guidelines for animal care and use.

### 2.3. Semen Processing and Storage Protocol

Fresh chicken eggs were surface-sterilized, and cracked to separate the yolks. The yolk membrane was punctured with a syringe to extract pure egg yolk, which was subsequently filtered through a 0.22 μm filter membrane, uniformly mixed, and prepared as the yolk supplementation component. Selected fresh semen with normal color and odor was preliminarily screened for motility and concentration using a computer-assisted sperm analysis system (CASAS, Minitube, Tiefenbach, Germany). Semen samples with a sperm concentration above 2 × 10^9^ cells/mL and sperm motility exceeding 80% were pooled to minimize individual variation and served as the sample for the subsequent experiments. Following the initial motility assessment, the pooled ram semen was diluted in prewarmed (25 °C) basal diluent. The basic extender was supplemented with 1% penicillin-streptomycin-gentamicin (PSG, Solaibao, Beijing, China) and 10% of the yolk-based component described above. The semen and diluent were mixed isothermally at a dilution ratio of 1:10 and the diluted samples were labeled properly.

The eight groups of diluted semen samples were then stored at 17 °C, and sperm motility was detected every 24 h to systematically evaluate the effects of the different diluents on semen preservation.

### 2.4. Assessment of Sperm Motility

A 20 μL aliquot was carefully collected from the middle layer of the well-mixed semen in each experimental group using a micropipette and transferred into a centrifuge tube. The sample was gradually restored to physiological temperature in a 37 °C water bath. After 1 min incubation at 37 °C, a 10 μL droplet was taken and placed onto a pre-warmed (37 °C) microscope slide to prepare a sample for microscopic examination. Sperm motility was assessed via CASAS, with 5 random fields of view observed, each containing no less than 200 spermatozoa to ensure analytical accuracy.

### 2.5. Assessment of Sperm Acrosome Integrity

The acrosome integrity of sperm was evaluated utilizing a fluorescein isothiocyanate-conjugated Peanut Agglutinin (PNA-FITC, Shanghai Haling Biotechnology Co., Ltd., Shanghai, China) kit and flow cytometry. The operational procedure was as follows: Semen samples were resuspended in a suitable volume of PBS and centrifuged at 1000 r/min for 5 min. The supernatant was discarded, and the washing step was repeated twice. The sperm concentration was adjusted to 1.2 × 10^7^ cells/mL. Subsequently, 100 μL of the semen suspension was transferred to a centrifuge tube, mixed with 200 μL of PNA-FITC staining solution, and incubated in the dark at room temperature for 20 min. After incubation, the mixture was centrifuged at 1000 r/min for 5 min. The supernatant was discarded, and the pellet was resuspended in 500 μL PBS. This washing step with PBS was performed twice. Ultimately, the sperm pellet was resuspended in 50 μL PBS for flow cytometry analysis. The flow cytometer (LSM T-PMT, ZEISS, Oberkochen, Germany) was set with an excitation wavelength of 488 nm and emission wavelengths of 530 nm and 630 nm. A minimum of 50,000 cells were analyzed for each sample.

### 2.6. Assessment of Sperm Plasma Membrane Integrity

Sperm plasma membrane integrity was evaluated using an Annexin V/PI detection kit (C0080, Solarbio, Beijing, China) combined with flow cytometry. The detailed procedure was as follows: the semen samples were centrifuged at 1000 r/min for 5 min, and the supernatant was discarded. The pellet was resuspended in 200 μL of PBS and centrifuged again. This washing step was repeated 2–3 times. The sperm pellet was then resuspended in 300 μL of 1× Binding Buffer, and the cell concentration was adjusted to 1 × 10^6^ cells/mL. Thereafter, 100 μL of sperm suspension was incubated with 5 μL of FITC-Annexin V stain, gently blended, and incubated in the dark at room temperature for 10 min, followed by the addition of 5 μL of PI staining solution and further incubation for 5 min under the same conditions. After staining, 500 μL of PBS was added to the mixture, followed by centrifugation at 1000 r/min to remove the supernatant. The washing process was repeated twice. Ultimately, the stained spermatozoa were resuspended in 400 μL 1× Binding Buffer, and 200 μL of the sample was used for flow cytometric analysis. The flow cytometer settings, including the excitation wavelength, were consistent with those described in Section 2.5, and at least 20,000 cells were analyzed per sample.

### 2.7. Statistical Analysis

Statistical analysis was performed utilizing SPSS software (version 27.0.1, IBM Corp., Armonk, NY, USA). All experiments were performed with at least three independent replicates. The results are presented as the mean ± standard deviation. A probability (*p*) value of <0.05 was defined as statistically significant, whereas *p* > 0.05 indicated no significant difference.

### 2.8. Sperm Metabolomics Analysis

Untargeted UPLC-MS metabolomics was performed on Mongolian ram spermatozoa (from the highest-motility group) at 0 h (H0), 48 h (H48), 96 h (H96), and 144 h (H144) of storage, with six replicates for each time point. Samples were centrifuged at 4 °C (3500 r/min, 5 min), washed twice with DPBS, and stored at −80 °C. Metabolomic analysis was performed by OE Biotech Co., Ltd. (Shanghai, China). LC-MS detection was carried out on a Waters UPLC-Q Exactive HF system (QE-HF, Thermo Fisher Scientific, Waltham, MA, USA) with an HSS T3 column (100 mm × 2.1 mm, 1.8 μm, Waters, Milford, MA, USA) at 45 °C. Mobile phases were 0.1% formic acid aqueous solution and acetonitrile, with a linear gradient elution at 0.35 mL/min and an injection volume of 5 μL. MS was operated in positive/negative switching mode, including full scan (*m*/*z* 70–1050, resolution 60,000) and MS/MS (resolution 15,000, collision energies 10/20/40 eV). Multivariate analyses (PCA, PLS-DA) were performed using the R packages ropls, with heatmaps and volcano plots generated by pheatmap and ggplot2, respectively.

## 3. Results

### 3.1. Preservation of Mongolian Sheep Sperm in Different Extenders at 17 °C

The sperm parameters of Mongolian sheep stored at 17 °C are presented in Table 2. Sperm motility: At 48 h of storage, no statistically significant differences were observed among all groups (*p* > 0.05). At 144 h, Groups 7 and 8 displayed significantly higher motility than Groups 1–6 (*p* < 0.05), whereas no significant difference was found between groups 7 and 8 (*p* > 0.05). The overall motility ranking was as follows: Group 8 > Group 7 > Group 6 > Group 5 > Group 4 > Group 3 > Group 1 > Group 2. Consequently, diluents from Groups 6, 7, and 8 were selected for the subsequent assessments of sperm plasma membrane integrity and acrosome integrity.

Sperm plasma membrane integrity: At multiple time points (24 h, 48 h, 96 h, 120 h, and 144 h), Group 8 maintained significantly higher integrity compared to the other groups (*p* < 0.05). The overall integrity ranking was Group 8 > Group 7 > Group 6.

Sperm acrosome integrity: At 24 h and 144 h, Group 6 showed markedly lower integrity than Groups 7 and 8 (*p* < 0.05), with no significant difference observed between Groups 7 and 8. At 96 h, significant differences were detected among all three groups. The general ranking was Group 8 > Group 7 > Group 6.

### 3.2. Compositional Characteristics of Differential Sperm Metabolites

**Figure 1:**
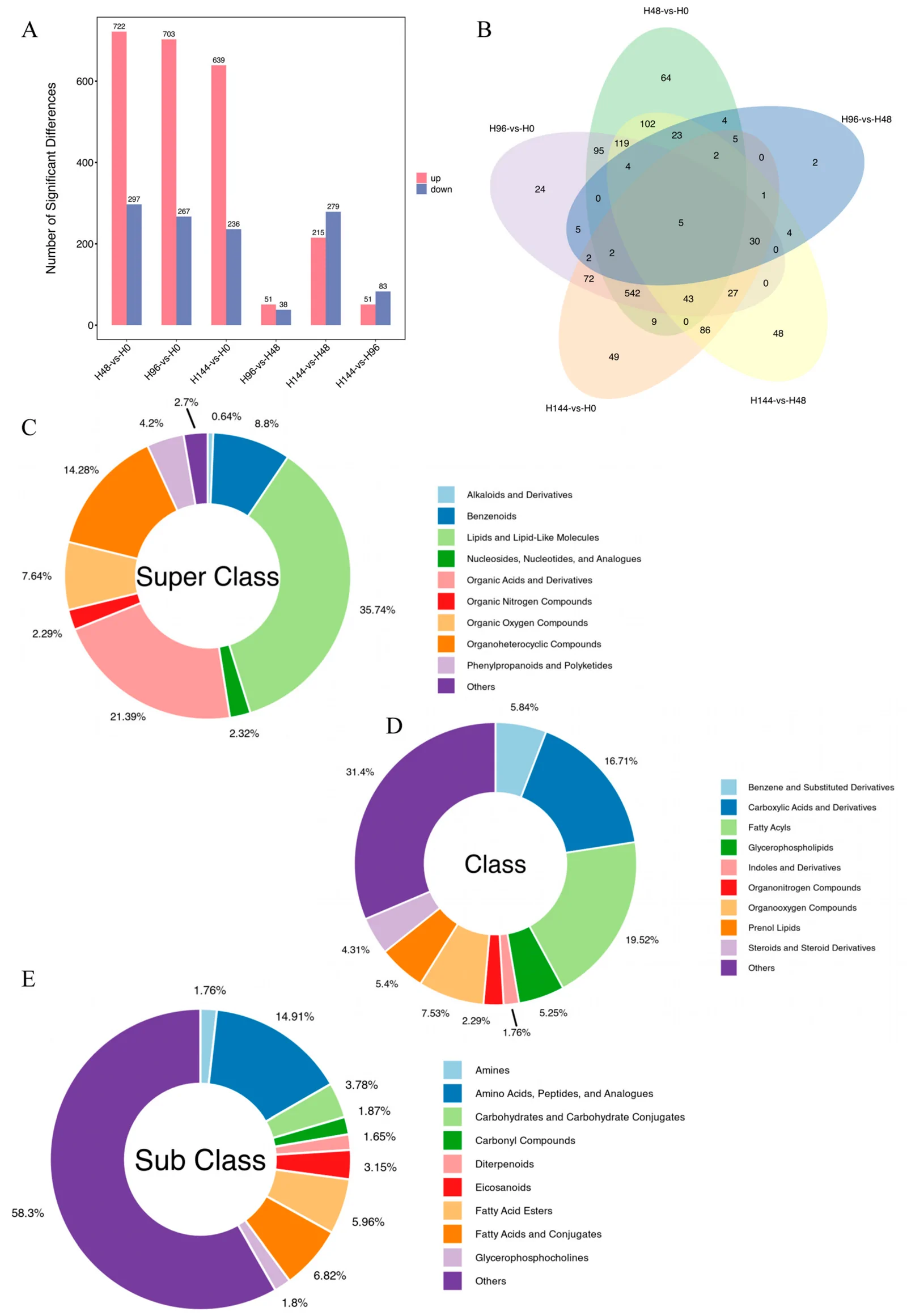
Metabolomic profiling of sperm at 17 °C. (**A**) The number of differentially metabolites at each storage time point compared to 0 h (up-regulated in red, down-regulated in blue). (**B**) A Venn diagram illustrating the overlap of differential metabolites among the comparisons (H48 vs. H0, H96 vs. H0, H144 vs. H0, H96 vs. H48, and H144 vs. H96). (**C**) A differential metabolite classification pie chart at the Super Class level. (**D**) A differential metabolite classification pie chart at the Class level. (**E**) A differential metabolite classification pie chart at the Sub Class level.

On this basis, the 2669 metabolites detected in all samples (including 350 at level 1, 734 at level 2, 234 at level 3, and 1351 at level 4) were subjected to further analysis. Differential metabolites were screened using the criteria of VIP > 1 and *p*-value < 0.05, with their quantitative distribution detailed in Figure 1A. A hierarchical classification analysis of the identified differential metabolites revealed that, at the Super Class level (Figure 1C), lipids and lipid-like molecules (35.74%) constituted the predominant fraction during sperm preservation, followed by organic acids and derivatives (21.39%) and organoheterocyclic compounds (14.28%). Collectively, these three major metabolite groups accounted for 71.41% of the total metabolites. At the subclass level (Figure 1D), fatty acids (19.52%) and carboxylic acids and derivatives (16.71%) were the two most abundant metabolite classes. At the subclass level (Figure 1E), amino acids, peptides, and their analogs (14.91%) represented the most abundant known category, trailed by fatty acids and their conjugates (6.82%) and fatty acid esters (5.96%).

### 3.3. Dynamic Changes in Differential Metabolites During Storage

To identify the core metabolites consistently altered across the entire process, Venn diagram analysis was performed on comparison groups at different time points. As shown in Figure 1B, five metabolites (spirotaccagenin, thiol-maleimide, 9-hydroxy-4-methoxypsoralen 9-glucoside, tezosentan, pyrrhoxanthinol) were shared by all five comparison groups (H48 vs. H0, H96 vs. H0, H96 vs. H48, H144 vs. H0, H144 vs. H48), which were identified as the most stable and central metabolic markers during sperm storage at 17 °C. On a broader scale, the three groups compared to the initial state (H48 vs. H0, H96 vs. H0, and H144 vs. H0) shared 593 common differential metabolites. Superclass analysis of these metabolites revealed that lipids and lipid-like molecules constituted the highest proportion at 48.6%.

**Figure 2:**
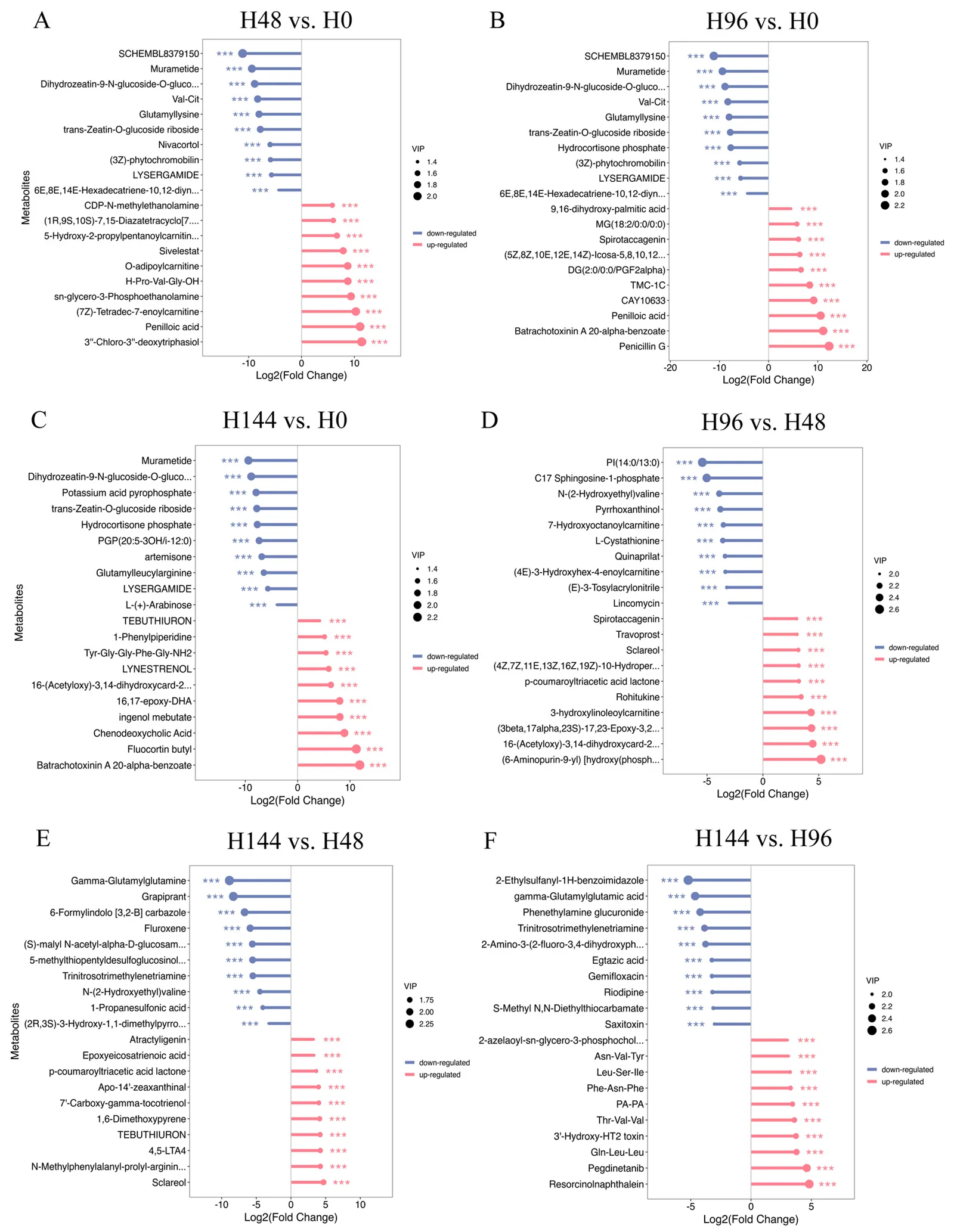
Analysis of differential metabolites at different semen storage stages based on non-targeted metabolomics. (**A**–**F**) The series of lollipop plots show significantly differential metabolites in pairwise comparisons between different storage stages. Specific comparison groups include (**A**) H48 vs. H0, (**B**) H96 vs. H0, (**C**) H144 vs. H0, (**D**) H96 vs. H48, (**E**) H144 vs. H48, and (**F**) H144 vs. H96. *** Indicates a *p*-value is less than 0.001 and greater than 0.0001. Some metabolite names were truncated in the plots for layout purposes, and their full names are provided as follows: Figure 2A includes Dihydrozeatin-9-N-glucoside-O-glucoside, 6E,8E,14E-Hexadecatriene-10,12-diynoic acid, (1R,9S,10S)-7,15-Diazatetracyclo[7.7.1.02,7.010,15]heptadeca-2,4-dien-6-one, and 5-Hydroxy-2-propylpentanoylcarnitine; Figure 2B includes Dihydrozeatin-9-N-glucoside-O-glucoside, 6E,8E,14E-Hexadecatriene-10,12-diynoic acid, and (5Z,8Z,10E,12E,14Z)-Icosa-5,8,10,12,14-pentaenoylcarnitine; Figure 2C includes Dihydrozeatin-9-N-glucoside-O-glucoside and 16-(Acetyloxy)-3,14-dihydroxycard-20(22)-enolide; Figure 2D includes (4Z,7Z,11E,13Z,16Z,19Z)-10-Hydroperoxydocosahexaenoic acid, (3beta,17alpha,23S)-17,23-Epoxy-3,29-dihydroxy-27-norlanosta-7,9(11)-diene-15,24-dione, 16-(Acetyloxy)-3,14-dihydroxycard-20(22)-enolide, (6-Aminopurin-9-yl) [hydroxy(phosphonooxy)phosphoryl] hydrogen phosphate, 2-Amino-3-(2-fluoro-3,4-dihydroxyphenyl)propanoic acid, and 2-Azelaoyl-sn-glycero-3-phosphocholine; Figure 2E includes (S)-Malyl N-acetyl-alpha-D-glucosaminide, 5-Methylthiopentyldesulfoglucosinolate, (2R,3S)-3-Hydroxy-1,1-dimethylpyrrolidin-1-ium-2-carboxylate, and N-Methylphenylalanyl-prolyl-arginine.

A comparative analysis of the 10 differential metabolites with the lowest *p*-values from both the up-regulated and down-regulated groups unveiled a sequence of metabolites demonstrating consistent dynamic alterations throughout the preservation process. Notably, Dihydrozeatin-9-N-glucoside-O-glucoside and trans-Zeatin-O-glucoside riboside were persistently and significantly down-regulated (VIP > 1.8) in the H48 vs. H0 (Figure 2A), H96 vs. H0 (Figure 2B), and H144 vs. H0 (Figure 2C) comparisons, suggesting rapid consumption during the initial preservation phase. As the intermediate storage stage (H96 vs. H48, Figure 2D), the metabolite C17 Sphingosine-1-phosphate, instrumental in sustaining cellular homeostasis, exhibited a down-regulated trend, whereas certain antioxidant-associated metabolites began to fluctuate. In the advanced preservation stage (H144 vs. H96, Figure 2F), pronounced depletion of gamma-glutamylglutamic acid and notable alterations in oxidative stress indicators, such as egtazic acid, were detected. A significant up-regulation of p-coumaroyltriacetic acid lactone and epoxyeicosatrienoic acid was observed over the entire late preservation period (H144 vs. H48, Figure 2E).

Further in-depth analysis of differential metabolites yielded two major findings: (1) Several key metabolites that are crucial for maintaining normal sperm function decreased as storage time extended. These metabolites included phosphatidylcholine (22:5/0:0, 22:6/0:0, 22:6-2OH (10S, 17)/2:0) and phosphatidylethanolamine (24:6/0:0), which are involved in membrane structure and lipid homeostasis; AT-Resolvin D1, Flavinat, S-(11-OH-9-deoxy-delta9, 12-PGD2)-glutathione and resveratrol, which form the antioxidant system; 5′-Guanylic acid, alpha-D-glucose, D-serine, and Estriol succinate, which participate in energy metabolism and mitochondrial function; signaling molecules that regulate physiological functions, including steroid hormone metabolites, prostaglandins, and 20-trifluoro-LTB4; as well as amino acids such as arginine and proline, and other osmoregulatory substances. (2) A variety of metabolites indicative of cellular damage exhibited marked elevation corresponding to prolonged storage duration. These included prostaglandin derivatives (e.g., 13,14-dihydro-15-keto-PGE2, prostaglandin B3) and hydroxy fatty acids (e.g., alpha-9,10-DiHODE, 17(S)-HETE), serving as markers for membrane lipid peroxidation and inflammation; polyunsaturated fatty acid derivatives, exemplified by oleoylcarnitine, reflecting aberrant fatty acid metabolism; ethyltestosterone and 7alpha-hydroxytestosterone, pointing to disorders in steroid hormone metabolism; and detoxification and metabolic waste, including phenol sulfate and glycocholic acid. Concurrently, substances linked to oxidative stress and DNA damage, such as 7-hydroxycannabidiol, also exhibited an upward trend.

### 3.4. Oxidative Stress and Lipid Metabolism Reprogramming Jointly Drive Sperm Metabolic Dysfunction

**Figure 3:**
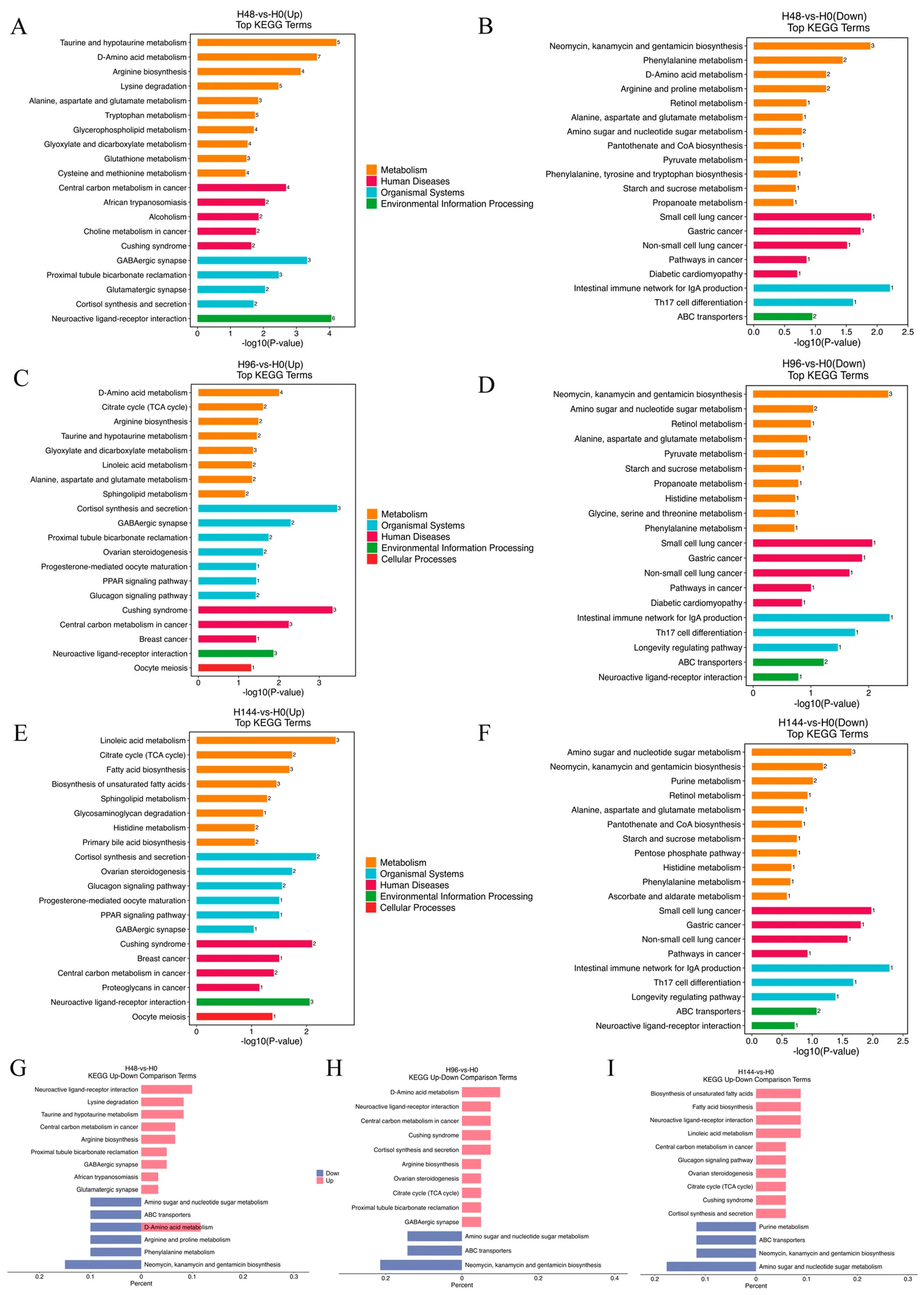
KEGG pathway enrichment analysis of the sperm metabolome across different preservation durations. (**A**,**C**,**E**) Bar plots of the top 20 significantly up-regulated KEGG pathways in sperm at 48 h (H48), 96 h (H96), and 144 h (H144), respectively, compared to the fresh sample baseline (H0). (**B**,**D**,**F**) Bar plots of the top 20 significantly down-regulated KEGG pathways at the corresponding time points (H48, H96, H144). (**G**–**I**) Comparative analysis of KEGG pathways via up–down comparison charts for H48, H96, and H144, respectively, vs. H0.

Under storage at 17 °C, sperm metabolites exhibited notable time-dependent alterations. During the initial preservation period (48 h), stress defense metabolic pathways such as Glutathione metabolism and Taurine and hypotaurine metabolism were significantly up-regulated (Figure 3A,G). Meanwhile, fundamental anabolic pathways, including pantothenate and CoA biosynthesis and pyruvate metabolism, began to decline (Figure 3B). During mid-term storage (96 h), metabolic patterns shifted. The citrate cycle, the Tricarboxylic Acid Cycle (TCA cycle), and linoleic acid metabolism were activated (Figure 3C,H). Meanwhile, numerous amino acid metabolic pathways continued to down-regulate (Figure 3D). In the late preservation stage (144 h), systemic metabolic depletion emerges, with substantial down-regulation of central antioxidant systems, including the pentose phosphate pathway and ascorbate and aldarate metabolism (Figure 3F). Simultaneously, lipid metabolism pathways, including fatty acid biosynthesis, biosynthesis of unsaturated fatty acids, and Peroxisome Proliferator-Activated Receptor (PPAR) signaling pathway, were highly activated (Figure 3E,I), whereas purine metabolism experienced severe disruption (Figure 3F). Specifically, this metabolic state was characterized by the depletion of protective metabolites such as phospholipid choline (22:5/0:0) and resveratrol, along with elevated levels of membrane lipid peroxidation-related metabolites, such as the prostaglandin derivative 13,14-dihydro-15-keto-PGE2 and oleoylcarnitine.

### 3.5. Positive Effects of Differential Metabolites on Sperm Preservation Quality

To verify the reliability of the above metabolomic findings and develop efficient preservation strategies, three antioxidant substances were selected for a combined intervention study based on the metabolomic analysis. The selection rationale was as follows: Firstly, resveratrol and its metabolites were notably down-regulated during preservation, indicating that the depletion of their endogenous levels might be closely correlated with the deterioration of sperm function. Consequently, resveratrol was given priority as a “metabolic replacement”. Secondly, vitamin E, a well-recognized classic lipid-soluble antioxidant, was selected as exogenous antioxidant protection. Thirdly, lycopene was incorporated owing to its potent ability to quench singlet oxygen and its distinctive protective role in lipid environments, with the aim of specifically targeting the central issue of “lipid peroxidation” identified in our study. The combined application of these three substances is anticipated to synergistically alleviate sperm oxidative damage during the preservation process through multiple pathways.

#### 3.5.1. Optimal Concentration Selection of Differential Metabolites

To explore the effects of resveratrol, lycopene, and vitamin E on sperm preserved at 17 °C, various types and concentrations of antioxidants were individually supplemented to the basic diluent (Group 8), and sperm kinematic parameters were evaluated. The findings revealed that the ideal concentrations of each antioxidant successfully prolonged the sperm preservation duration to 7 days. (1) Among the four tested Resveratrol concentrations (shown in Table 3), the 25 μM concentration group exhibited the best preservation performance based on the same indicators. Specifically, at 168 h, this group displayed 54.6% sperm motility, 54.01 μm/s average path velocity, and 81.66 μm/s curvilinear velocity, all significantly exceeding those in the 0, 10, and 40 μM groups (*p* < 0.05). (2) Lycopene at a particular concentration markedly improved sperm motility. As demonstrated in Table 4, in comparison to the control group without supplementation (0 μM), the 5 μM treatment group exhibited the highest motility (55.75%) and progressive motility at 168 h, which were notably higher than those of the control groups (*p* < 0.05). Nevertheless, no statistically significant differences were identified between the 5 μM group and the other two treatment groups. With regard to kinematic parameters, both the average path velocity (57.07 μm/s) and straight-line velocity (47.56 μm/s) in the 5 μM group were significantly higher than those in all other groups (*p* < 0.05). Notably, even at the early preservation stage (24 h), the average path velocity (83.75 μm/s) and straight-line velocity (73.78 μm/s) in the 5 μM group remained at the initial levels and were significantly superior to those of the control and other concentration groups (*p* < 0.05). Additionally, this group maintained optimal sperm linearity and lateral head displacement amplitude throughout storage. (3) Among the four tested concentrations of vitamin E, the 1.5 μM group exhibited the highest motility (53.37%) at 168 h, which was significantly higher than that in the 0 μM and 0.5 μM groups *(p* < 0.05). Although its motility was greater than that in the 3 μM group, the difference was not statistically significant. Furthermore, at this concentration (1.5 μM), sperm kinematic parameters attained the peak levels, as outlined in Table 5, and this advantage remained stable even following 24 h of preservation. Combined with the results of sperm plasma membrane and acrosome integrity (shown in Table 6), it is evident that all three antioxidants, at their respective optimal concentrations, exert favorable protective effects on sheep semen preservation.

#### 3.5.2. Optimal Combination Selection of Differential Metabolites

Various antioxidant combinations effectively sustained the motility of Mongolian sheep sperm for up to 7 days at 17 °C. Notably, Group D (resveratrol + lycopene + vitamin E) exhibited the best overall preservation efficacy (Table 7). During the late storage stage (72–168 h), sperm motility and forward movement rate in Group D were significantly higher than or comparable to those of other groups (sperm motility after 168 h: Group D 55.05% vs. Group A 53.72%, Group B 53.10%, Group C 52.51%). Regarding movement speed, the average path velocity (137.55 μm/s) and straight-line velocity (127.55 μm/s) of Group D at 24 h were significantly greater than those of the other groups, and it still retained this velocity advantage at 168 h. Moreover, sperm in Group D displayed the highest sperm movement linearity and the optimal maintenance of head lateral swing amplitude, suggesting superior movement and morphological stability. When evaluating acrosome and plasma membrane integrity (Table 8), Group D exerted the most favorable effect in preserving the structural integrity and functional activity of sperm throughout the 168 h preservation period. In terms of membrane integrity, the values of Group D remained the highest at all time points, particularly at the endpoint of preservation (168 h), reaching 69.06%, significantly surpassing Group B (67.25%). With regard to acrosome integrity, Group D also demonstrated stable performance, with an acrosome integrity rate of 87.20% at 168 h, significantly higher than that of Group C (86.00%) (*p* < 0.05), indicating a superior structural protection capacity.

In summary, the combined application of the three antioxidants (Group D) demonstrated prominent advantages in prolonging sperm preservation duration and maintaining motility quality.

## 4. Discussion

This study systematically uncovered the metabolic mechanisms driving functional deterioration of Mongolian sheep sperm stored at 17 °C by the integration of sperm quality evaluation and non-targeted metabolomics analysis. Our findings not only confirmed the notable effect of storage duration on sperm quality but, more crucially, illustrated a dynamic, multi-phase metabolic reprogramming process at the molecular level.

Firstly, phenotypic screening verified the effects of eight different diluent formulations on sperm preservation. Among them, the eighth formulation, which employed fructose and lactose as dual sugar sources, exhibited the best performance in maintaining sperm motility, plasma membrane integrity, and acrosome integrity (Table 2). Importantly, this study highlighted the beneficial effects of fructose and lactose in particular formulations, contrasting with the conclusion of Mu et al. [24] favoring sucrose. This inconsistency probably arises from interactions with other elements in the diluent, such as buffering systems and antioxidants. This implies that the protective effects of sugars are not independent but contingent on their overall chemical environment [25].

This study utilized non-targeted metabolomics to disclose, for the initial time, the temporal metabolic alterations in Mongolian sheep sperm during storage at 17 °C. Lipid metabolism reprogramming constituted the most notable adaptive change, with lipids and lipid-like molecules accounting for 35.74% of all differential metabolites, dominated by fatty acids (19.52%) and carboxylic acids and derivatives (16.71%), alongside abundant glycerophospholipids. (Figure 1C,D). In boar sperm preserved at 17 °C, lipids and lipid-like molecules were also identified as the predominant category of differential metabolites, and with lysophosphatidylcholine (lysoPC(20:3)) recognized as a biomarker of preservability [26]. In bovine sperm, organic acids and their derivatives constitute the predominant metabolic class, and the compositional profiles of specific fatty acids—such as palmitic acid and oleic acid—are closely associated with fertilizing capacity [27]. Collectively, these findings confirm that the balanced membrane lipid metabolism is a key determinant of sperm preservation efficacy.

Temporal analysis of differential metabolites revealed that Dihydrozeatin-9-N-glucoside-O-glucoside and trans-Zeatin-O-glucoside riboside were persistently and markedly down-regulated from the initial phase of preservation. This suggested that the early depletion of cytokinin-like substances may impair the sperm’s capacity to sustain homeostasis [28]. These observations were consistent with the role of such metabolites as active reserves in plant systems. As the preservation duration extended, metabolic disturbances intensified gradually. The up-regulation of C17 sphingosine-1-phosphate observed during the mid-preservation phase signified the activation of the sphingolipid signaling pathway, potentially representing a cellular protective response to preservation stress [29]. Nonetheless, in the late preservation phase, substantial depletion of Gamma-Glutamylglutamic acid denoted the disruption of glutathione metabolism, and notable alterations in oxidative stress indicators such as Egtazic acid substantiated the detrimental impact of reactive oxygen species accumulation [30]. Ultimately, the widespread down-regulation of p-coumaroyltriacetic acid lactone and Epoxyeicosatrienoic acid reflected the extensive dysregulation of the metabolic network, indicating collapse of the antioxidant system and irreversible damage to sperm function [31]. This evolution pattern has also been observed in liquid-preserved boar sperm, suggesting that the temporal evolution of the sperm metabolic network may follow a conserved intrinsic pattern under preservation stress [32].

KEGG analysis revealed up-regulation of glutathione metabolism and taurine and hypotaurine metabolism during the initial 48 h of storage (Figure 3A). Previous studies have confirmed that boar sperm can synthesize glutathione (GSH) from sulfur-containing amino acids (cysteine, glutamine, and serine) to scavenge reactive oxygen species (ROS); nevertheless, intracellular GSH levels, antioxidant enzyme activities (e.g., glutathione peroxidase), and redox homeostasis in sperm gradually decline with prolonged storage, requiring exogenous supplementation for maintenance [33,34]. In contrast, we observed significant early up-regulation of this pathway in ram sperm at 48 h; however, this finding alone cannot support the assumption that endogenous GSH levels in ram sperm would continuously decline with extended preservation time. As the storage duration extended to 96 h, the glutathione metabolism and taurine and hypotaurine metabolism pathways that were up-regulated at 48 h were no longer significantly enriched, whereas the TCA cycle and linoleic acid metabolism were markedly activated, suggesting a metabolic shift from antioxidant defense toward lipid-dependent oxidative phosphorylation (Figure 3C,H). The up-regulation shows that sperm strived to sustain ATP supply by boosting fatty acid β-oxidation and the TCA cycle [35,36,37]. Interestingly, in boar sperm during liquid preservation, the decline in total ATP after 1 day of storage mainly stemmed from reduced mitochondrial oxidative phosphorylation (OXPHOS), whereas glycolytically derived ATP (glycoATP) remained stable [38]. By day 3, a more pronounced metabolic shift from OXPHOS toward glycolysis was observed, accompanied by an approximate 15% reduction in ATP production [39]. These findings indicate that the compensatory response of boar sperm to energy crisis relies primarily on glycolysis rather than fatty acid oxidation, which differs from the mid-phase metabolic shift observed in ram sperm toward TCA cycle and lipid oxidation. At the late storage stage (144 h, Figure 3E,I), the pentose phosphate pathway and ascorbate and aldarate metabolism were substantially down-regulated. This suggests diminished antioxidant metabolic capacity of spermatozoa for NADPH generation and maintenance of reduced glutathione, thereby potentially compromising their buffering capacity against sustained oxidative stress [40,41,42]. Meanwhile, pathways including fatty acid biosynthesis, biosynthesis of unsaturated fatty acids, and PPAR signaling were highly activated. This suggests the possible redistribution of lipid metabolism in spermatozoa at later stage of preservation, characterized by adjustments in lipid composition and adaptation of energy metabolism. [43,44,45]. The PPAR signaling pathway is crucial for maintaining sperm energy balance. It promotes fatty acid oxidation to generate energy, while simultaneously activating antioxidant genes (such as GPX4) to alleviate oxidative damage. Additionally, it regulates apoptosis to maintain sperm numbers [46]. Concurrently, impaired purine metabolism not only disrupted energy supply (e.g., ATP, GTP) but also facilitated accumulation of its degradation product, hypoxanthine. Under the influence of oxidases, hypoxanthine can generate a large amount of reactive oxygen species, thereby further exacerbating oxidative damage [47,48].

This research also identified a sequence of distinct metabolite changes matching the above modifications in metabolic pathways. The continuous reduction in antioxidants including resveratrol, along with the rise in metabolites associated with membrane lipid peroxidation, such as prostaglandin derivatives and oleoylcarnitine, offers direct evidence for heightened oxidative damage. Among them, lycopene can protect sperm DNA by inhibiting lipid peroxidation, while resveratrol boosts antioxidant capacity through activating the SIRT1 pathway [16]. The decline in 5′-guanylic acid and alpha-D-glucose suggests diminished mitochondrial ATP synthesis efficiency and compromised glycolysis [49]. On the other hand, metabolite elevations signify compensatory reactions and metabolic dysfunction. For example, the increased carnitine-bound fatty acid indicates a compensatory surge in mitochondrial β-oxidation [50]. Collectively, these alterations suggest that storage at 17 °C disrupts sperm metabolic homeostasis, triggering a series of compensatory mechanisms.

Based on the above metabolomics findings and validation analyses, resveratrol, vitamin E, and lycopene were selected for a combined intervention study. This design was guided by multiple considerations: Firstly, the marked down-regulation of resveratrol during preservation strongly indicates that its endogenous depletion constitutes a pivotal event in the dysregulation of the sperm metabolic network. Supplementation with resveratrol as a “metabolic substitute” aims not only to restore its direct antioxidant function but also to explore whether it can reprogram downstream signaling pathways and metabolic homeostasis. Secondly, vitamin E, a well-established fat-soluble antioxidant, serves as a reference to evaluate the efficacy of exogenous antioxidants, and establish a defined protective baseline in composite interventions [51]. Finally, the inclusion of lycopene was selected as a targeted choice based on the core research finding that lipid metabolism reprogramming and membrane lipid peroxidation are central to preservation-induced damage. Its remarkable capacity to quench singlet oxygen is anticipated to directly intervene and stabilize the sperm plasma membrane, which is rich in polyunsaturated fatty acids, thereby mitigating the cascade amplification of oxidative stress at the source [52,53]. In summary, the combination of these three substances is not merely a simple additive mixture; it constructs a multi-level, synergistic defense system covering endogenous metabolites replenishment (resveratrol), classic antioxidant defense (vitamin E), and targeted membrane lipid protection (lycopene). This system is anticipated to achieve synergistic effects by targeting different nodes of oxidative damage, thereby offering a novel and theoretically well-founded strategy to alleviate sperm preservation-induced damage.

For this purpose, the function of these substances in sperm preservation and their optimal effective concentrations were initially confirmed through exogenous supplementation with varying concentrations of antioxidants, namely resveratrol, lycopene, and vitamin E. The experimental results revealed that supplementation with 25 μM resveratrol, 5 μM lycopene, or 1.5 μM vitamin E could effectively mitigate sperm quality decline during preservation, extend valid sperm preservation duration to 7 days, and sustain motility above 50% (Table 4, Table 5 and Table 6). Subsequently, the combined supplementation of the three antioxidants yielded the optimal protective outcome (sperm motility reached 55.05%), suggesting a synergistic effect in their antioxidant function. This offers a reliable combination strategy for constructing an efficient sperm preservation system.

It is worth noting that, despite the promising preservation performance of this combined formulation, several limitations of this study should be acknowledged. First, pooled semen samples were used during the initial extender screening phase to minimize individual variation and enable unbiased comparison of formulations. While this approach is suitable for screening, it does not capture inter-animal differences in semen quality or genetic merit. Therefore, our findings reflect average population-level preservation outcomes and cannot directly support individual animal genetic selection or conservation strategies. Second, although the optimized formulation (diluent 8 combined with resveratrol, lycopene, and vitamin E) effectively preserved sperm quality parameters in vitro, fertility outcomes—such as conception or lambing rates—were not evaluated in this study. The correlation between in vitro sperm quality and in vivo fertilizing capacity under our experimental conditions requires further investigation through artificial insemination trials. We regard this as an essential prerequisite before field application of this formulation in breeding programs.

Despite these limitations, our findings deliver both mechanistic insights and practical guidance for optimizing liquid preservation of ram semen at 17 °C, with the ternary antioxidant combination representing a promising formulation strategy.

## 5. Conclusions

The optimal protocol for preserving Mongolian sheep sperm at 17 °C employs a base diluent containing fructose (1%, *w*/*v*) and lactose (1%, *w*/*v*), supplemented with the ternary combination of resveratrol (25 μM), lycopene (5 μM), and vitamin E (1.5 μM).

## Figures and Tables

**Table 1:** Formula for semen extender.

	Component (g) ^1^	Glucose	Sucrose	Fructose	Lactose	Tris	Citric Acid
Group							
1		2				3	1.6
2			2			3	1.6
3				2		3	1.6
4					2	3	1.6
5		1	1			3	1.6
6		1		1		3	1.6
7		1			1	3	1.6
8				1	1	3	1.6

^1^ The basal diluent components were sourced as follows: Glucose (G5500, Sigma-Aldrich, St. Louis, MO, USA), sucrose (S8271, Solaibao, Beijing, China), fructose (F0127, Sigma-Aldrich, St. Louis, MO, USA), lactose (D8310, Solaibao, Beijing, China), Tris (10812846001, Sigma-Aldrich, St. Louis, MO, USA), and citric acid (PHR1071, Sigma-Aldrich, St. Louis, MO, USA).

**Table 2:** Sperm parameters of Mongolian sheep at 17 °C.

	Time	Storage Time/h					
Group		24	48	72	96	120	144
Sperm motility/%	1	81.50 ± 2.39 ^d^	76.46 ± 2.12 ^a^	70.03 ± 6.62 ^d^	65.16 ± 2.80 ^c^	53.57 ± 0.74 ^d^	23.27 ± 1.08 ^e^
	2	82.33 ± 1.89 ^d^	74.41 ± 2.64 ^a^	68.26 ± 3.12 ^d^	61.51 ± 5.17 ^c^	25.43 ± 4.69 ^c^	0 ± 0 ^f^
	3	83.32 ± 1.49 ^cd^	77.10 ± 5.40 ^a^	71.67 ± 2.13 ^cd^	67.90 ± 2.17 ^b^	56.06 ± 3.47 ^c^	28.28 ± 2.48 ^d^
	4	84.59 ± 2.56 ^bcd^	80.40 ± 2.05 ^a^	74.53 ± 2.3 ^bc^	69.27 ± 1.26 ^b^	62.52 ± 2.33 ^b^	36.26 ± 2.88 ^c^
	5	86.30 ± 4.38 ^abc^	75.50 ± 26.60 ^a^	75.96 ± 4.45 ^b^	69.87 ± 1.95 ^b^	64.38 ± 6.13 ^ab^	37.68 ± 0.81 ^c^
	6	87.30 ± 2.46 ^ab^	80.49 ± 2.63 ^a^	79.64 ± 2.28 ^a^	74.83 ± 2.19 ^a^	65.02 ± 2.38 ^ab^	41.39 ± 6.64 ^b^
	7	87.70 ± 3.71 ^ab^	82.28 ± 1.66 ^a^	80.47 ± 1.20 ^a^	76.53 ± 4.16 ^a^	65.93 ± 5.25 ^ab^	44.33 ± 3.83 ^a^
	8	88.36 ± 4.11 ^a^	83.13 ± 2.85 ^a^	81.27 ± 3.40 ^a^	77.2 ± 2.82 ^a^	66.94 ± 2.08 ^a^	46.63 ± 1.59 ^a^
Sperm membrane integrity rate/%	6	77.71 ± 1.14 ^b^	76.57 ± 0.90 ^b^	73.80 ± 0.69 ^c^	70.83 ± 1.58 ^b^	68.73 ± 0.83 ^b^	65.29 ± 2.09 ^b^
	7	79.41 ± 0.51 ^ab^	77.05 ± 0.57 ^b^	74.62 ± 0.18 ^b^	72.29 ± 0.64 ^ab^	70.10 ± 0.71 ^b^	66.55 ± 1.37 ^ab^
	8	80.12 ± 1.10 ^a^	78.61 ± 0.47 ^a^	77.62 ± 0.34 ^a^	74.03 ± 0.31 ^a^	72.54 ± 0.70 ^a^	68.98 ± 0.12 ^a^
Sperm acrosome integrity rate/%	6		93.00 ± 0.70 ^b^		90.89 ± 0.45 ^c^		88.45 ± 1.08 ^b^
	7		95.22 ± 0.37 ^a^		92.03 ± 0.61 ^b^		91.04 ± 0.40 ^a^
	8		96.02 ± 0.48 ^a^		93.77 ± 0.35 ^a^		92.03 ± 0.47 ^a^

Identical superscript letters for different groups within the same column indicate that there is no significant difference between groups (*p* > 0.05), while the superscript letters that are different indicate significant differences (*p* < 0.05). Comparisons were not performed among data within the same row. Data are presented as the mean ± SD (*n* = 3).

**Table 3:** Sperm motility in different concentrations of resveratrol diluent at 17 °C.

Motion Parameters	Concentration/μM	Storage Time/h				
		0	24	72	120	168
Sperm motility/%	0	84.90 ± 1.85 ^a^	81.70 ± 1.44 ^a^	71.43 ± 0.15 ^b^	55.42 ± 0.41 ^b^	49.84 ± 1.36 ^c^
	10	84.90 ± 1.85 ^a^	81.47 ± 1.79 ^a^	73.07 ± 1.25 ^ab^	59.88 ± 1.04 ^ab^	49.48 ± 0.62 ^c^
	25	84.90 ± 1.85 ^a^	82.10 ± 1.22 ^a^	77.21 ± 1.37 ^a^	65.90 ± 1.16 ^a^	54.60 ± 0.95 ^a^
	40	84.90 ± 1.85 ^a^	81.77 ± 1.91 ^a^	74.87 ± 1.42 ^ab^	61.20 ± 1.36 ^ab^	51.24 ± 1.04 ^b^
Forward motion/%	0	54.83 ± 5.81 ^a^	38.52 ± 7.31 ^b^	33.23 ± 2.67 ^b^	30.26 ± 3.36 ^b^	27.90 ± 0.57 ^b^
	10	54.83 ± 5.81 ^a^	40.99 ± 6.37 ^b^	38.23 ± 2.16 ^ab^	33.82 ± 2.87 ^ab^	29.77 ± 2.77 ^a^
	25	54.83 ± 5.81 ^a^	48.33 ± 4.21 ^a^	42.71 ± 2.35 ^a^	35.99 ± 4.31 ^a^	30.44 ± 4.75 ^a^
	40	54.83 ± 5.81 ^a^	45.77 ± 3.79 ^ab^	36.55 ± 1.62 ^b^	32.16 ± 3.93 ^ab^	29.78 ± 5.77 ^a^
Average path speed/μm/s	0	100.42 ± 8.95 ^a^	77.66 ± 6.93 ^ab^	56.41 ± 8.21 ^b^	49.93 ± 4.23 ^c^	51.38 ± 2.77 ^ab^
	10	100.42 ± 8.95 ^a^	63.84 ± 2.11 ^b^	60.70 ± 5.67 ^ab^	44.66 ± 5.81 ^d^	48.72 ± 2.01 ^b^
	25	100.42 ± 8.95 ^a^	80.46 ± 0.64 ^a^	68.71 ± 9.35 ^a^	59.79 ± 2.77 ^a^	54.01 ± 4.25 ^a^
	40	100.42 ± 8.95 ^a^	77.81 ± 6.52 ^ab^	68.19 ± 4.54 ^a^	52.65 ± 4.61 ^b^	49.21 ± 5.21 ^b^
Linear motion speed/μm/s	0	84.90 ± 8.02 ^a^	64.08 ± 7.63 ^b^	37.32 ± 6.86 ^b^	43.71 ± 6.08 ^b^	38.63 ± 5.77 ^b^
	10	84.90 ± 8.02 ^a^	60.22 ± 5.38 ^b^	59.24 ± 5.67 ^a^	42.89 ± 8.47 ^b^	39.12 ± 7.36 ^b^
	25	84.90 ± 8.02 ^a^	71.18 ± 5.63 ^a^	61.65 ± 8.68 ^a^	49.79 ± 5.51 ^a^	43.02 ± 9.94 ^a^
	40	84.90 ± 8.02 ^a^	71.43 ± 4.46 ^a^	59.51 ± 6.18 ^a^	44.73 ± 6.45 ^b^	38.45 ± 7.24 ^b^
Curved motion velocity/μm/s	0	164.40 ± 7.72 ^a^	135.19 ± 5.62 ^a^	81.68 ± 2.24 ^c^	84.23 ± 4.27 ^b^	70.38 ± 4.91 ^b^
	10	164.40 ± 7.72 ^a^	116.89 ± 5.32 ^b^	99.73 ± 6.13 ^b^	81.71 ± 4.04 ^b^	72.39 ± 2.02 ^b^
	25	164.40 ± 7.72 ^a^	136.73 ± 4.29 ^a^	107.21 ± 2.04 ^a^	97.06 ± 2.31 ^a^	81.66 ± 7.94 ^a^
	40	164.40 ± 7.72 ^a^	135.81 ± 3.51 ^a^	93.36 ± 2.14 ^b^	84.87 ± 1.33 ^b^	71.85 ± 6.81 ^b^
Linearity of the motion path/%	0	52.11 ± 3.37 ^a^	47.72 ± 5.49 ^b^	40.91 ± 7.42 ^b^	41.71 ± 1.97 ^ab^	38.74 ± 4.04 ^ab^
	10	52.11 ± 3.37 ^a^	51.28 ± 2.77 ^a^	44.46 ± 2.22 ^ab^	39.80 ± 4.18 ^ab^	38.27 ± 2.31 ^ab^
	25	52.11 ± 3.37 ^a^	51.66 ± 3.83 ^a^	48.87 ± 7.60 ^a^	43.92 ± 3.78 ^a^	41.85 ± 4.14 ^a^
	40	52.11 ± 3.37 ^a^	47.36 ± 5.79 ^b^	48.66 ± 1.81 ^a^	38.68 ± 0.65 ^b^	36.76 ± 3.46 ^b^
Lateral displacement amplitude of the head/μm	0	5.20 ± 0.80 ^a^	4.93 ± 1.01 ^b^	4.57 ± 0.79 ^a^	3.68 ± 1.39 ^ab^	3.54 ± 0.28 ^ab^
	10	5.20 ± 0.80 ^a^	5.02 ± 0.78 ^a^	4.65 ± 0.67 ^a^	3.48 ± 0.38 ^b^	3.92 ± 0.41 ^a^
	25	5.20 ± 0.80 ^a^	5.03 ± 0.78 ^a^	4.72 ± 0.19 ^a^	3.99 ± 1.39 ^a^	3.91 ± 0.30 ^a^
	40	5.20 ± 0.80 ^a^	5.01 ± 2.81 ^a^	4.57 ± 1.12 ^a^	3.53 ± 0.85 ^b^	3.68 ± 0.35 ^ab^

If the superscript letters for different groups within the same column are identical, no significant difference exists between groups (*p* > 0.05); if the superscript letters differ, a significant difference exists (*p* < 0.05). Data within the same row are not compared. Data are presented as the mean ± SD (n = 3).

**Table 4:** Sperm motility in different concentrations of lycopene diluent at 17 °C.

Motion Parameters	Concentration/μM	Storage Time/h				
		0	24	72	120	168
Sperm motility/%	0	85.03 ± 1.70 ^a^	83.60 ± 1.85 ^ab^	76.87 ± 1.63 ^a^	62.46 ± 0.10 ^b^	51.20 ± 0.21 ^b^
	2	85.03 ± 1.70 ^a^	83.30 ± 1.39 ^ab^	75.06 ± 1.69 ^a^	63.99 ± 0.36 ^b^	52.79 ± 1.22 ^ab^
	5	85.03 ± 1.70 ^a^	84.97 ± 1.53 ^a^	76.54 ± 1.51 ^a^	66.70 ± 1.01 ^a^	55.75 ± 1.30 ^a^
	10	85.03 ± 1.70 ^a^	82.90 ± 1.56 ^b^	72.20 ± 2.64 ^b^	63.26 ± 1.78 ^b^	52.55 ± 1.86 ^ab^
Forward motion/%	0	47.53 ± 3.67 ^a^	41.37 ± 0.81 ^b^	36.36 ± 6.63 ^b^	34.62 ± 5.91 ^ab^	27.83 ± 3.58 ^b^
	2	47.53 ± 3.67 ^a^	43.23 ± 1.38 ^ab^	38.11 ± 4.93 ^ab^	33.00 ± 7.45 ^b^	30.32 ± 6.50 ^ab^
	5	47.53 ± 3.67 ^a^	45.23 ± 1.29 ^a^	40.15 ± 1.13 ^a^	35.76 ± 7.12 ^a^	33.32 ± 6.76 ^a^
	10	47.53 ± 3.67 ^a^	40.39 ± 0.09 ^b^	38.52 ± 4.88 ^ab^	34.79 ± 7.81 ^ab^	32.21 ± 7.77 ^ab^
Average path speed/μm/s	0	83.36 ± 6.32 ^a^	69.30 ± 1.72 ^b^	61.96 ± 8.32 ^a^	54.31 ± 2.13 ^b^	50.44 ± 7.40 ^b^
	2	83.36 ± 6.32 ^a^	70.61 ± 2.16 ^b^	56.93 ± 9.21 ^b^	49.96 ± 7.26 ^c^	49.14 ± 4.97 ^b^
	5	83.36 ± 6.32 ^a^	83.75 ± 7.29 ^a^	60.81 ± 5.33 ^a^	59.99 ± 5.77 ^a^	57.07 ± 1.67 ^a^
	10	83.36 ± 6.32 ^a^	77.51 ± 5.66 ^ab^	60.74 ± 7.02 ^a^	52.17 ± 2.05 ^b^	48.77 ± 2.16 ^b^
Linear motion speed/μm/s	0	73.26 ± 3.70 ^a^	52.50 ± 3.69 ^c^	36.36 ± 7.21 ^b^	36.54 ± 3.36 ^c^	34.76 ± 1.94 ^c^
	2	73.26 ± 3.70 ^a^	56.63 ± 4.83 ^b^	37.26 ± 5.46 ^b^	41.34 ± 5.86 ^b^	33.92 ± 5.44 ^c^
	5	73.26 ± 3.70 ^a^	73.78 ± 3.89 ^a^	45.09 ± 4.74 ^a^	51.97 ± 3.19 ^a^	47.56 ± 1.19 ^a^
	10	73.26 ± 3.70 ^a^	58.49 ± 3.94 ^b^	37.65 ± 6.76 ^b^	42.03 ± 6.63 ^b^	38.59 ± 4.87 ^b^
Curved motion velocity/μm/s	0	136.11 ± 5.58 ^a^	131.40 ± 3.34 ^b^	86.05 ± 4.16 ^c^	88.66 ± 3.40 ^b^	78.67 ± 6.55 ^c^
	2	136.11 ± 5.58 ^a^	143.02 ± 4.15 ^a^	93.97 ± 3.79 ^b^	98.06 ± 5.89 ^a^	87.66 ± 6.59 ^b^
	5	136.11 ± 5.58 ^a^	141.27 ± 5.37 ^a^	99.91 ± 7.83 ^a^	98.64 ± 8.94 ^a^	98.11 ± 6.06 ^a^
	10	136.11 ± 5.58 ^a^	134.88 ± 3.97 ^b^	83.71 ± 3.19 ^c^	90.09 ± 3.72 ^b^	88.78 ± 8.26 ^b^
Linearity of the motion path/%	0	50.52 ± 4.48 ^a^	48.25 ± 3.92 ^ab^	43.93 ± 5.69 ^b^	42.19 ± 2.06 ^b^	40.03 ± 0.08 ^a^
	2	50.52 ± 4.48 ^a^	47.02 ± 2.65 ^ab^	45.32 ± 2.94 ^b^	42.23 ± 2.66 ^b^	39.40 ± 0.73 ^b^
	5	50.52 ± 4.48 ^a^	52.53 ± 4.35 ^a^	49.97 ± 2.91 ^a^	44.43 ± 4.55 ^a^	41.42 ± 2.47 ^a^
	10	50.52 ± 4.48 ^a^	46.25 ± 1.76 ^b^	41.87 ± 3.38 ^c^	44.27 ± 2.65 ^a^	41.91 ± 2.88 ^a^
Lateral displacement amplitude of the head/μm	0	4.94 ± 0.06 ^a^	4.72 ± 0.08 ^ab^	4.35 ± 0.28 ^ab^	3.89 ± 0.53 ^b^	3.78 ± 0.41 ^b^
	2	4.94 ± 0.06 ^a^	4.46 ± 0.10 ^b^	4.15 ± 0.10 ^ab^	4.19 ± 0.33 ^ab^	3.80 ± 0.23 ^b^
	5	4.94 ± 0.06 ^a^	4.56 ± 0.09 ^b^	4.62 ± 0.39 ^a^	4.29 ± 0.97 ^a^	4.12 ± 0.22 ^a^
	10	4.94 ± 0.06 ^a^	4.80 ± 0.04 ^a^	3.72 ± 1.08 ^b^	4.41 ± 0.26 ^a^	4.08 ± 0.11 ^a^

If the superscript letters for different groups within the same column are identical, no significant difference exists between groups (*p* > 0.05); if the superscript letters differ, a significant difference exists (*p* < 0.05). Data within the same row are not compared. Data are presented as the mean ± SD (n = 3).

**Table 5:** Sperm motility in different concentrations of Vitamin E diluent at 17 °C.

Motion Parameters	Concentration/μM	Storage Time/h				
		0	24	72	120	168
Sperm motility/%	0	85.47 ± 0.12 ^a^	83.17 ± 2.78 ^a^	72.23 ± 1.58 ^b^	65.73 ± 2.77 ^a^	46.22 ± 1.02 ^b^
	0.5	85.47 ± 0.12 ^a^	83.63 ± 3.3 ^a^	74.50 ± 1.25 ^a^	62.61 ± 1.48 ^b^	47.47 ± 2.22 ^b^
	1.5	85.47 ± 0.12 ^a^	84.30 ± 2.07 ^a^	75.71 ± 1.12 ^a^	65.90 ± 1.24 ^a^	53.37 ± 0.97 ^a^
	3	85.47 ± 0.12 ^a^	84.53 ± 3.80 ^a^	72.57 ± 1.25 ^b^	62.60 ± 1.36 ^b^	51.20 ± 1.06 ^a^
Forward motion/%	0	40.60 ± 1.73 ^a^	30.73 ± 3.88 ^b^	26.35 ± 3.76 ^c^	25.41 ± 1.73 ^b^	20.98 ± 1.24 ^b^
	0.5	40.60 ± 1.73 ^a^	35.80 ± 6.91 ^a^	26.87 ± 5.55 ^c^	25.48 ± 3.86 ^b^	21.24 ± 1.67 ^b^
	1.5	40.60 ± 1.73 ^a^	37.37 ± 1.05 ^a^	35.30 ± 1.14 ^a^	30.77 ± 1.07 ^a^	27.45 ± 2.73 ^a^
	3	40.60 ± 1.73 ^a^	35.30 ± 5.01 ^a^	30.24 ± 2.69 ^b^	29.07 ± 5.66 ^a^	22.25 ± 6.32 ^b^
Average Path Speed/μm/s	0	80.54 ± 6.57 ^a^	71.10 ± 3.78 ^b^	59.97 ± 7.33 ^b^	50.89 ± 2.01 ^b^	44.71 ± 5.44 ^b^
	0.5	80.54 ± 6.57 ^a^	78.15 ± 5.89 ^a^	57.58 ± 4.16 ^b^	50.21 ± 7.20 ^ab^	45.16 ± 8.27 ^b^
	1.5	80.54 ± 6.57 ^a^	78.49 ± 7.11 ^a^	66.16 ± 1.37 ^a^	58.78 ± 4.43 ^a^	55.48 ± 0.91 ^a^
	3	80.54 ± 6.57 ^a^	78.73 ± 5.09 ^a^	55.83 ± 5.35 ^c^	51.24 ± 9.03 ^b^	40.87 ± 0.54 ^c^
Linear motion speed/μm/s	0	61.00 ± 7.91 ^a^	59.82 ± 5.89 ^a^	47.71 ± 3.83 ^a^	37.67 ± 6.31 ^b^	35.3 ± 4.25 ^a^
	0.5	61.00 ± 7.91 ^a^	60.54 ± 2.31 ^a^	43.87 ± 4.76 ^b^	36.80 ± 5.69 ^b^	32.29 ± 2.61 ^b^
	1.5	61.00 ± 7.91 ^a^	60.70 ± 8.89 ^a^	47.75 ± 4.59 ^a^	43.09 ± 5.51 ^a^	36.89 ± 4.09 ^a^
	3	61.00 ± 7.91 ^a^	60.75 ± 5.31 ^a^	42.94 ± 3.11 ^ab^	38.65 ± 7.32 ^b^	30.11 ± 8.54 ^b^
Curved motion velocity/μm/s	0	149.04 ± 5.01 ^a^	109.55 ± 6.81 ^c^	91.14 ± 5.88 ^b^	83.14 ± 7.21 ^c^	70.38 ± 24.9 ^c^
	0.5	149.04 ± 5.01 ^a^	112.69 ± 2.12 ^c^	93.79 ± 8.17 ^b^	88.99 ± 2.23 ^b^	78.73 ± 5.23 ^b^
	1.5	149.04 ± 5.01 ^a^	124.79 ± 4.45 ^b^	103.12 ± 9.14 ^a^	96.45 ± 6.11 ^a^	89.38 ± 6.26 ^a^
	3	149.04 ± 5.01 ^a^	132.77 ± 4.31 ^a^	94.27 ± 4.81 ^b^	86.92 ± 5.77 ^b^	74.25 ± 1.25 ^bc^
Linearity of the motion path/%	0	50.14 ± 0.33 ^a^	46.59 ± 0.63 ^b^	38.67 ± 5.53 ^b^	37.61 ± 5.77 ^b^	32.78 ± 3.41 ^b^
	0.5	50.14 ± 0.33 ^a^	47.03 ± 1.99 ^ab^	40.26 ± 6.37 ^b^	39.19 ± 2.13 ^a^	35.04 ± 1.73 ^b^
	1.5	50.14 ± 0.33 ^a^	48.87 ± 3.55 ^a^	46.27 ± 6.39 ^a^	40.90 ± 6.14 ^a^	39.08 ± 7.61 ^a^
	3	50.14 ± 0.33 ^a^	47.09 ± 2.27 ^ab^	43.30 ± 1.83 ^ab^	39.74 ± 1.16 ^a^	33.18 ± 4.91 ^b^
Lateral displacement amplitude of the head/μm	0	5.48 ± 0.53 ^a^	4.86 ± 0.80 ^ac^	4.59 ± 0.88 ^b^	3.88 ± 0.51 ^b^	3.47 ± 0.41 ^b^
	0.5	5.48 ± 0.53 ^a^	5.47 ± 0.39 ^b^	4.92 ± 0.41 ^a^	4.04 ± 0.59 ^ab^	3.97 ± 0.04 ^a^
	1.5	5.48 ± 0.53 ^a^	5.46 ± 0.61 ^b^	4.73 ± 0.51 ^a^	4.32 ± 0.46 ^a^	3.99 ± 0.51 ^a^
	3	5.48 ± 0.53 ^a^	5.24 ± 0.50 ^a^	4.57 ± 0.28 ^b^	4.30 ± 0.24 ^a^	3.67 ± 0.97 ^ab^

If the superscript letters for different groups within the same column are identical, no significant difference exists between groups (*p* > 0.05); if the superscript letters differ, a significant difference exists (*p* < 0.05). Data within the same row are not compared. Data are presented as the mean ± SD (n = 3).

**Table 6:** Integrity of sperm plasma membrane and acrosome in extenders supplemented with different antioxidants.

	Time	Storage Time/h				
Type		0	24	72	120	168
Sperm membrane integrity rate/%	0	79.82 ± 1.45 ^a^	76.36 ± 1.34 ^c^	74.32 ± 1.19 ^c^	70.36 ± 0.91 ^ab^	66.38 ± 1.80 ^cd^
	Resveratrol	79.82 ± 1.45 ^a^	77.51 ± 1.03 ^bc^	75.70 ± 2.01 ^ab^	70.74 ± 0.94 ^ab^	67.34 ± 0.95 ^bc^
	Lycopene	79.82 ± 1.45 ^a^	77.75 ± 1.54 ^bc^	75.41 ± 0.86 ^ab^	68.88 ± 1.15 ^b^	66.80 ± 1.30 ^cd^
	Vitamin E	79.82 ± 1.45 ^a^	76.38 ± 1.57 ^c^	74.26 ± 1.05 ^c^	67.62 ± 1.66 ^b^	65.87 ± 1.07 ^d^
Sperm acrosome integrity rate/%	0	91.30 ± 1.07 ^a^	89.39 ± 0.51 ^a^	88.42 ± 1.33 ^b^	85.01 ± 1.43 ^d^	84.24 ± 0.76 ^d^
	Resveratrol	91.30 ± 1.07 ^a^	90.01 ± 1.74 ^a^	88.54 ± 1.35 ^ab^	86.24 ± 1.21 ^cd^	85.88 ± 1.08 ^bc^
	Lycopene	91.30 ± 1.07 ^a^	89.97 ± 1.67 ^a^	88.92 ± 1.19 ^ab^	86.31 ± 1.69 ^cd^	85.80 ± 1.83 ^c^
	Vitamin E	91.30 ± 1.07 ^a^	89.99 ± 1.64 ^a^	88.60 ± 1.38 ^b^	86.08 ± 1.04 ^cd^	85.51 ± 1.67 ^c^

If the superscript letters for different groups within the same column are identical, no significant difference exists between groups (*p* > 0.05); if the superscript letters differ, a significant difference exists (*p* < 0.05). Data within the same row are not compared. Data are presented as the mean ± SD (n = 3).

**Table 7:** Changes in Mongolian sheep sperm viability during storage in diluents supplemented with different antioxidant mixtures.

Motion Parameters	Group	Storage Time/h				
		0	24	72	120	168
Sperm motility/%	A	85.30 ± 1.94 ^a^	83.74 ± 0.57 ^a^	81.30 ± 0.25 ^ab^	70.10 ± 0.34 ^ab^	53.72 ± 0.89 ^ab^
	B	85.30 ± 1.94 ^a^	82.95 ± 0.03 ^a^	77.74 ± 0.92 ^c^	65.74 ± 0.88 ^b^	53.10 ± 0.89 ^ab^
	C	85.30 ± 1.94 ^a^	83.00 ± 0.79 ^a^	80.08 ± 0.17 ^ab^	67.75 ± 0.38 ^b^	52.51 ± 0.70 ^b^
	D	85.30 ± 1.94 ^a^	83.70 ± 0.99 ^a^	82.74 ± 0.66 ^a^	74.75 ± 0.92 ^a^	55.05 ± 0.82 ^a^
Forward motion/%	A	64.74 ± 2.41 ^a^	59.92 ± 2.73 ^a^	51.24 ± 1.93 ^b^	48.04 ± 2.62 ^a^	38.28 ± 2.14 ^ab^
	B	64.74 ± 2.41 ^a^	61.44 ± 2.00 ^a^	53.14 ± 2.68 ^a^	47.94 ± 1.74 ^b^	38.94 ± 1.88 ^ab^
	C	64.74 ± 2.41 ^a^	52.64 ± 2.73 ^b^	51.84 ± 2.32 ^b^	48.54 ± 2.58 ^a^	37.34 ± 1.80 ^b^
	D	64.74 ± 2.41 ^a^	61.46 ± 2.97 ^a^	53.64 ± 1.08 ^a^	48.37 ± 1.05 ^a^	39.92 ± 1.10 ^a^
Average path speed/μm/s	A	129.04 ± 6.76 ^a^	123.85 ± 1.61 ^b^	88.58 ± 2.74 ^b^	70.79 ± 2.02 ^b^	58.94 ± 1.06 ^ab^
	B	129.04 ± 6.76 ^a^	125.62 ± 1.91 ^b^	91.41 ± 2.10 ^a^	72.37 ± 2.19 ^ab^	58.45 ± 2.03 ^b^
	C	129.04 ± 6.76 ^a^	120.44 ± 2.81 ^b^	90.81 ± 1.05 ^ab^	75.74 ± 1.31 ^a^	59.75 ± 2.32 ^ab^
	D	129.04 ± 6.76 ^a^	137.55 ± 2.46 ^a^	91.06 ± 1.83 ^a^	75.71 ± 2.01 ^a^	61.15 ± 2.38 ^a^
Linear motion speed/μm/s	A	118.93 ± 5.19 ^a^	103.42 ± 1.50 ^ab^	79.14 ± 2.00 ^ab^	58.16 ± 1.15 ^b^	48.18 ± 1.12 ^b^
	B	118.93 ± 5.19 ^a^	106.65 ± 2.66 ^ab^	79.64 ± 1.46 ^ab^	61.44 ± 1.45 ^ab^	48.85 ± 2.00 ^ab^
	C	118.93 ± 5.19 ^a^	84.91 ± 2.74 ^b^	76.12 ± 1.40 ^b^	61.52 ± 2.91 ^ab^	48.54 ± 1.33 ^ab^
	D	118.93 ± 5.19 ^a^	127.55 ± 1.31 ^a^	82.32 ± 2.03 ^a^	62.28 ± 2.97 ^a^	49.35 ± 1.22 ^a^
Curved motion velocity/μm/s	A	204.45 ± 5.33 ^a^	188.32 ± 2.11 ^ab^	146.31 ± 2.08 ^b^	108.32 ± 2.32 ^b^	92.32 ± 1.96 ^ab^
	B	204.45 ± 5.33 ^a^	192.05 ± 2.88 ^a^	148.94 ± 1.10 ^ab^	108.81 ± 2.92 ^b^	93.78 ± 2.76 ^a^
	C	204.45 ± 5.33 ^a^	181.57 ± 2.18 ^b^	144.05 ± 2.49 ^b^	108.69 ± 2.25 ^b^	90.82 ± 2.21 ^b^
	D	204.45 ± 5.33 ^a^	192.71 ± 2.79 ^a^	150.61 ± 2.69 ^a^	110.65 ± 2.69 ^a^	94.93 ± 1.66 ^a^
Linearity of the motion path/%	A	54.54 ± 2.61 ^a^	53.35 ± 1.94 ^a^	50.61 ± 1.99 ^ab^	47.41 ± 1.23 ^ab^	45.58 ± 1.32 ^ab^
	B	54.54 ± 2.61 ^a^	53.56 ± 1.21 ^a^	48.85 ± 1.99 ^b^	46.15 ± 1.23 ^b^	44.42 ± 1.28 ^b^
	C	54.54 ± 2.61 ^a^	53.80 ± 2.23 ^a^	51.21 ± 1.82 ^ab^	48.06 ± 2.09 ^a^	44.86 ± 1.56 ^ab^
	D	54.54 ± 2.61 ^a^	53.88 ± 2.94 ^a^	52.59 ± 1.53 ^a^	48.91 ± 1.30 ^a^	46.89 ± 2.80 ^a^
Lateral displacement amplitude of the head/μm	A	6.68 ± 1.04 ^a^	6.22 ± 0.43 ^a^	5.41 ± 0.71 ^a^	4.78 ± 0.03 ^ab^	4.21 ± 0.62 ^ab^
	B	6.68 ± 1.04 ^a^	6.15 ± 0.43 ^a^	5.38 ± 0.56 ^a^	4.79 ± 0.41 ^ab^	4.29 ± 0.73 ^ab^
	C	6.68 ± 1.04 ^a^	6.32 ± 0.56 ^a^	5.37 ± 0.70 ^a^	4.56 ± 0.85 ^b^	4.12 ± 0.26 ^b^
	D	6.68 ± 1.04 ^a^	6.39 ± 0.11 ^a^	5.58 ± 1.00 ^a^	5.02 ± 0.54 ^a^	4.35 ± 0.97 ^a^

Combination Groups: Group A: 25 μM resveratrol + 5 μM lycopene; Group B: 5 μM lycopene + 1.5 μM vitamin E; Group C: 25 μM resveratrol + 1.5 μM vitamin E; Group D: 25 μM resveratrol + 5 μM lycopene + 1.5 μM vitamin E. Within the same column, data points labeled with the same lowercase letter indicate no significant difference (*p* > 0.05), while different letters indicate a significant difference (*p* < 0.05). Data within the same row are not compared. Data are presented as the mean ± SD (n = 3).

**Table 8:** Integrity of sperm plasma membrane and acrosome in extenders supplemented with different antioxidant combinations.

	Time	Storage Time/h				
Type		0	24	72	120	168
Sperm membrane integrity rate/%	A	79.82 ± 1.45 ^a^	78.92 ± 1.24 ^a^	75.61 ± 1.51 ^ab^	71.81 ± 1.18 ^a^	68.44 ± 2.09 ^ab^
	B	79.82 ± 1.45 ^a^	78.22 ± 1.02 ^ab^	75.23 ± 1.63 ^ab^	69.96 ± 0.34 ^ab^	67.25 ± 1.68 ^bc^
	C	79.82 ± 1.45 ^a^	77.79 ± 0.90 ^bc^	75.10 ± 1.72 ^bc^	69.92 ± 0.67 ^ab^	68.32 ± 0.94 ^ab^
	D	79.82 ± 1.45 ^a^	79.30 ± 1.31 ^a^	76.11 ± 1.71 ^a^	72.36 ± 1.07 ^a^	69.06 ± 1.54 ^a^
Sperm acrosome integrity rate/%	A	91.30 ± 1.07 ^a^	90.44 ± 0.50 ^a^	89.18 ± 0.92 ^a^	87.16 ± 87.31 ^ab^	86.98 ± 0.96 ^ab^
	B	91.30 ± 1.07 ^a^	90.49 ± 1.43 ^a^	89.05 ± 0.85 ^ab^	87.01 ± 87.55 ^ab^	86.81 ± 86.56 ^ab^
	C	91.30 ± 1.07 ^a^	90.42 ± 1.42 ^a^	89.04 ± 1.66 ^ab^	86.87 ± 87.55 ^bc^	86.00 ± 1.24 ^bc^
	D	91.30 ± 1.07 ^a^	90.87 ± 1.74 ^a^	89.67 ± 0.51 ^a^	87.95 ± 1.85 ^a^	87.20 ± 1.31 ^a^

Combination Groups: Group A: 25 μM resveratrol + 5 μM lycopene; Group B: 5 μM lycopene + 1.5 μM vitamin E; Group C: 25 μM resveratrol + 1.5 μM vitamin E; Group D: 25 μM resveratrol + 5 μM lycopene + 1.5 μM vitamin E. Within the same column, data points labeled with the same lowercase letter indicate no significant difference (*p* > 0.05), while different letters indicate a significant difference (*p* < 0.05). Data within the same row are not compared. Data are presented as the mean ± SD (n = 3).

## Data Availability

The findings presented in this research are accessible from the corresponding author upon sub-mission of a reasonable request. Metabolomics data have been submitted to the Zenodo database at https://doi.org/10.5281/zenodo.21872792.

## Abbreviations

The following abbreviations are used in this manuscript:

TCA cycle	Tricarboxylic Acid Cycle
PPAR signaling pathway	Peroxisome Proliferator-Activated Receptor signaling pathway
KEGG	Kyoto Encyclopedia of Genes and Genomes
